# What do other people think he deserves? Social influence on utilization of mitigating information regarding a violent offender’s unfortunate life history

**DOI:** 10.1371/journal.pone.0291729

**Published:** 2023-11-17

**Authors:** Michael J. Gill, Sinenhlanhla P. Zungu

**Affiliations:** Department of Psychology, Lehigh University, Bethlehem, PA, United States of America; Polytechnic Institute of Coimbra: Instituto Politecnico de Coimbra, PORTUGAL

## Abstract

The blameworthiness of an offender is often discussed in groups. Yet, the research literature overwhelmingly examines individuals assessing blameworthiness in isolation. To address this gap in the literature, the present study examines group deliberations about blameworthiness, with a particular focus on how group deliberations impact utilization of mitigating information about an offender’s unfortunate life history. Participants from introductory psychology courses at a U.S. university were placed in groups of two or three and each group also included a confederate who followed a script. Groups were randomly assigned to one of four conditions. In one condition (deed only), groups learned only about the offender’s heinous crimes. In the three remaining conditions, participants also received a historicist narrative regarding how the offender’s unfortunate history deformed his moral character. These conditions differed in terms of the confederate’s arguments: Neutral arguments, arguments to ignore the narrative, or arguments to give great weight to the narrative. Results showed that the historicist narrative was particularly effective at reducing outrage and increasing compassion when the confederate argued for its utilization. The reduction in outrage mediated a reduction in spiteful punitiveness toward the offender. Interestingly, the confederate who urged fellow deliberators to ignore the historicist narrative had no impact on outrage or compassion. We also examined mediation of the impact of historicist narratives on outrage and compassion. We found that when the confederate remained neutral the impact of historicist narratives on outrage and compassion was mediated via diminished perceptions of the offender’s control of self-formation. This mirrors what is typically found in prior work focused on individual judgments. In contrast, when the confederate argued that great weight should be given to the narrative, reductions in outrage were mediated via diminished perceptions of offender freedom of action. This pattern of mediation is not typically found but has been found in one previous study where participants received social encouragement to mitigate blame. Results are discussed in terms of how social influence might alter the inferences draw from historicist narratives. Suggestions for future research on social influence in the context of blame are presented.

## Introduction

How much should we blame our sister for forgetting our birthday? How much should we blame our son’s teacher for a chaotic classroom that impedes learning? How much should we blame an offender with an unfortunate life history for his violent crimes?

Although these offenses are quite different, they share at least one thing in common: The offender’s blameworthiness is likely to be examined in deliberations with others. That is, siblings will discuss how much to blame their sister; parents will discuss how much to blame their son’s teacher; and a jury will discuss how much to blame a violent offender. Although it seems obvious that assessments of blameworthiness often involve group deliberations, the blame literature comprises almost exclusively studies of individuals making blame judgments in isolation from others. Whereas this work provides important insights into the mechanisms by which people assess blameworthiness, it overlooks a common feature of the social context of blaming.

The present study joins a small number of prior studies aiming to fill this gap in the literature. We will focus on how deliberations among peers impact the utilization of mitigating information regarding a violent offender who committed a double homicide. The mitigating information will be a *historicist narrative* that explains how the offender’s character was damaged by an unfortunate life history [1–4]. Going beyond prior work, we will insert a confederate—posing as a peer—into the blame deliberations, with the confederate making scripted arguments that either challenge or encourage utilization of the mitigating historicist narrative information. Our key question is *whether* and, if so, *why* the confederate’s arguments impact participants’ personal thoughts about the offender’s blameworthiness.

It is important to understand factors that influence utilization of offender history information. One reason for this is that such information is commonly presented during actual criminal justice proceedings, especially when the offender has committed violent acts that might warrant a death sentence [5–7]. In such contexts, the willingness to mitigate blame based on an offender’s unfortunate history is a matter of life and death for the offender. More broadly, people engage in historicist thinking about offenders in all sorts of social domains and such thinking can reduce gratuitous, counterproductive punitiveness [1–4].

Prior to describing our study, we will clarify what we mean by “blame,” discuss the literature on historicist narratives and moderators of their utilization, and review the literature on social influence in group deliberations regarding criminal offenders.

## What do we mean by blame?

Moral philosophers have spent much energy striving to define the concept of blame [8]. A degree of consensus has emerged around the view that blame is, at its core, angry, hostile emotions directed toward a norm-violating target. One writer has suggested that any conceptualization of blame that does not have such emotion at the core “leaves the blame out of blame” [9; pg. 349]. Following from this analysis, we define blame as “a negative affective response—characterized by *other-condemning emotions* [10] (anger, contempt, disgust)—that is triggered by a norm violation and which targets the violator” [11; pg. 2]. In our studies below, we will assess compassion for the offender in addition to blame of the offender. This approach of separately measuring both angry and compassionate emotions is common in the blame literature [1, 4, 12].

### From abuse history to historicist narratives: Understanding why offender history mitigates blame

How is blame impacted when a perceiver learns about an offender’s unfortunate life history? Perhaps surprisingly, much prior work has suggested that people either completely ignore such information [13–15] or give it little weight in their judgments [16, 17]. These findings led Stevenson to lament that it is “sobering that child abuse appears not to be used as a mitigating factor” [18; pg. 346]. In one illustrative study that inspired the present work, Stevenson, Bottoms, and Diamond [19] examined how evidence regarding an offender’s unfortunate history was framed in discussions among mock jurors. The most common framing was that the history information should be ignored. In short, prior work has produced a surprising lack of evidence for a mitigating impact of offender history information. One exception appears to be cases in which a history of abuse leads one to kill one’s abuser. In that case, blame is mitigated presumably because the murder is considered justified–it’s understandable that a victim would kill her brutal abuser [20–23].

Gill and Cerce [1] argued that the null results described above are misleading. They suggested that the null results come from studies which assumed that *perceptions of prior suffering* per se would mitigate blame. The studies cited above cast doubt on this assumption. Yet, it might still be the case that offender history information can mitigate blame, but the mechanism might involve something other than perceived suffering. In line with this possibility, Gill and Cerce proposed that information about an unfortunate history would effectively mitigate blame when the information is “woven into a narrative that prompts the conclusion, ‘No wonder he turned out like he did!’” [pg. 363]. They coined the phrase *historicist narratives* to refer to story-like accounts that explain how an offender’s character was warped by powerful formative experiences. The crucial feature of historicist narratives, then, is that they *explain character formation* rather than *highlight prior suffering*.

Gill and Cerce [1] described six experiments that tested their ideas. In one experiment, participants learned about an office bully, James, who deliberately humiliates his subordinates. All participants learned about James’s awful behavior, but only some received a historicist narrative regarding how James’s father physically and emotionally abused him, leading James to believe that bullying is “the way of the world.” Next, participants made blame ratings (e.g., *I feel moral anger*). They also rated their perceptions on several potential mediators drawn from the blame literature: Perceived suffering (e.g., *James must have cried a lot as a little boy*); perceived intentionality (e.g., *James wants to humiliate others*); perceived freedom of action (e.g., *James could choose to STOP bullying*); and perceived control of self-formation (e.g., *James had free will in terms of initially BECOMING a bully*). Results across the studies indicated that historicist narratives consistently reduced blame and that diminished perceptions of control of self-formation—i.e., the offender did not self-create his own character—consistently mediated their mitigating impact. Interestingly, consistent with the argument above, perceptions of offender suffering were uncorrelated with blame.

### Historicist narratives can vary in their effectiveness

Prior research also suggests that the extent to which information about an unfortunate history mitigates blame can vary widely. For example, as noted above, some work from the criminal justice literature suggests that people often try to persuade others to ignore information about an offender’s unfortunate history [19]. Other work from that literature suggests that people will mitigate blame based on such information, but only if the information is paired with a scientifically-grounded theoretical rationale—e.g., early abuse breaks the social bonds that are necessary for positive development—justifying its use [24]. There is also evidence of greater mitigation when the unfortunate history is described as lasting for a long time [25]. Finally, other work in the criminal justice literature suggests that information about an unfortunate history has a larger mitigating effect among people who support the death penalty because those who oppose the death penalty support compassionate approaches to punishment regardless of offender history [26].

Similarly, within the historicist narrative research tradition—i.e., the social psychology rather than criminal justice literature—it has been shown that, although historicist narratives sometimes have a large mitigating effect [1], other times they are discounted for a variety of reasons. For example, Gill and Pizzuto [27] found that White people who blamed Black people for *societal*-level outcomes (e.g., racial disparities in education, crime, etc.) completely ignored an *individual*-level historicist narrative regarding a violent Black offender (e.g., his dad taught him that “being a man” required violence). In contrast, they utilized that same individual-level historicist narrative to mitigate blame of a violent White offender. In a different context, Gill and Thalla [2] showed that historicist narratives are less effective for blame mitigation when the offender shares his own narrative. This effect occurred because people expect self-aware offenders—who understand how their life history negatively impacted them—to have deeper comprehension of the wrongness of their own offending. Thus, they should know better than to harm others and this renders them more blameworthy.

Finally, Gill & Ungson [3] showed that utilization of historicist narratives was moderated by motivation to blame. In their Study 4, for example, participants read about a double homicide. Blame motivation was manipulated by asking some participants to play the role of prosecutor and others to play the role of defender. Furthermore, some participants learned only about the crime whereas others also learned a historicist narrative regarding the offender’s brutal upbringing. Next, participants were asked to present arguments that supported either harshness (for prosecutors) or mercy (for defenders). Then, participants were told that the role play portion of the study was over and that the researchers were interested in their *personal opinions* about the offender. Results indicated that those who had previously been defenders showed a stronger mitigating effect of the historicist narrative than did those who had been prosecutors. Mediational evidence suggested a process of self-persuasion: Defenders blamed the offender less than prosecutors in the historicist narrative condition because, *when playing the role of defender*, they had argued that the offender’s unfortunate history lowered his freedom of action. Note that, in contrast to the work above, this work suggests that historicist narratives sometimes—perhaps when perceivers receive external prompting to mitigate blame—reduce perceived freedom of action. This mediating role of freedom of action will become important later in this article.

Although our focus is on mitigating information regarding an offender’s unfortunate history, there is also an extensive literature concerning other types of mitigating information. One well-studied other type of mitigating information is biological narratives, which assert that an offender’s bad actions are caused by a biological abnormality (e.g., genes that foster violent behavior, mental disability). Some evidence suggests that biological narratives lead to greater certainty that mitigation is warranted than do historicist narratives [3, 28, 29], whereas other work suggests that biological narratives have complex implications that can lead either to mitigation or to aggravation of blame and punishment [30, 31]. Clearly, given the malleability of effects involving biological narratives, it would be worthwhile to study how social influence moderates whether and how they are utilized. Doing so, however, is beyond the scope of the present article.

### Deliberating about blameworthiness

Nearly all the work reviewed thus far has focused on individuals assessing blameworthiness in isolation from others. One contribution of our study below is to examine social influence during group deliberations regarding blameworthiness. Because of our focus on such group deliberations, here we review the small number of studies that focused on such deliberations.

Sommers [32] had participants learn about the case of a Black male accused of sexual assault. Participants deliberated in mock jury groups of six. The mock juries were either all White or included two Black participants (and four White). After watching a video that presented the case information, the mock juries deliberated. All but one group either reached a “not guilty” verdict or failed to achieve unanimity. Thus, verdicts could not be meaningfully analyzed. Aspects of the deliberation process, however, could be analyzed. Results indicated that, compared to all-White juries, racially diverse juries deliberated for longer, discussed a larger number of case facts, verbalized a smaller number of inaccuracies, were more likely to correct each other’s inaccuracies, were more likely to mention race, and were less likely object to race being mentioned. These differences were largely due to changes in the behavior of White jurors (rather than to Black jurors behaving differently than White jurors). Thus, social influence was evident: The mere presence of Black jurors made White jurors more thoughtful in their information processing. As noted, however, this did not translate into different verdicts.

Lynch and Haney [33] explored the role of emotion during mock jury deliberations. Each mock jury viewed a videotape that presented a homicide case. The prosecution presented several aggravating factors (prior record, lack of remorse) and the defense presented several mitigating factors (severe abuse during childhood, drug and alcohol problems). Perpetrator and victim race (Black, White) were both manipulated. After receiving all pertinent information, jurors engaged in deliberations. Analyses of the deliberations showed that anger and hostility toward the defendant were the most common emotions expressed. Compassion based on the abuse he had suffered was also fairly common. In terms of social influence, the results suggested that expressions of anger and hostility were treated as “normal” and required no justification. In contrast, expressions of compassion were sometimes condemned as “too emotional” by other jurors. Lynch and Haney presented some sample transcripts in which racial bias seemed to be evident in social influence processes—e.g., a juror arguing against compassion for a Black defendant—but they did not provide any statistical analysis showing that such arguments were made more frequently for Black than for White defendants or that such arguments statistically mediated more punitive responses to Black defendants in final verdicts. The clearest evidence in this study is for attempted social influence, with jurors stigmatizing compassion presumably to steer others toward punitiveness. Notably, our study below will examine the effectiveness of such peer arguments in persuading others.

Diamond, Murphy, and Rose [34] focused on the accuracy with which jurors applied relevant law during their deliberations. In actual court cases, the relevant law is communicated directly to the jury in the form of jury instructions. How accurately are such instructions followed? To examine this, Diamond et al. used data from the Arizona Jury Project, which included videotapes of deliberations from fifty actual juries. Analysis of the deliberations revealed that 17% of comments were about the laws described in the jury instructions. Juror utilization of that information was accurate nearly 80% of the time. Social influence was evident in a variety of ways. First, jurors contributed to accuracy by reading the relevant law out loud to their peers. Second, jurors impaired accuracy when they misunderstood the relevant law and passed their misunderstanding on to the group. Interestingly, however, positive social influence was evident in the fact that these errors were corrected by other jurors nearly half the time. Overall, Diamond et al. interpret the evidence as suggesting that jurors do a surprisingly good job in comprehending and applying the law—much better than some earlier lab-based work had suggested—and that social influence plays a key role in this strong performance. Indeed, Diamond et al. conclude that pessimistic views of juror comprehension are rooted in lab-based studies that focus on individuals in isolation from others.

Finally, in a study alluded to above, Stevenson, Bottoms, and Diamond [19] had mock juries watch a video that portrayed a re-enactment of the sentencing phase of a capital trial. The defendant had been convicted of murder and the jury was tasked with deciding whether he deserved the death penalty or something less. The deliberations were coded by the research team and particular attention was paid to how the mock jurors discussed the mitigating factors of abuse history and alcoholism. Results revealed that although jurors were more likely to argue that child abuse should be used as a mitigating factor (*The conditions of his childhood threw him into this*) than as an aggravating factor (*Maybe the damn kid deserved to be beat by his dad*), the most common juror argument was to simply ignore the abuse information (*Not every abused person ends up killing someone*). An identical pattern was found for alcoholism (*Booze is no excuse*). Like Lynch and Haney (2015), this study does not provide evidence regarding social influence but rather reveals attempted social influence (i.e., jurors’ efforts to persuade others). Our study below can be seen as a follow-up to this work because we directly examine whether (and why) deliberators are affected by arguments from their peers to either ignore or to give great weight to a mitigating factor.

In short, some studies suggest that group deliberations improve the accuracy or thoroughness of information processing [32, 34]. Other work shows that group members actively try to persuade others to be more or less harsh [19, 33]. Of the studies in this latter category, none provides evidence regarding the effectiveness of these persuasive efforts. Our study aims to provide such evidence.

## The present experiment

### Ethics statement

All studies were approved by Lehigh University’s Institutional Review Board (Proposal ID: 1149682–15). Participants signed an IRB-approved consent form prior to participating. No information was collected that would enable identification of any individual research participant during or after the study. Data are available at: Gill & Zungu - Open Science Framework.

The study below will examine the impact of historicist narratives on blame of a violent offender. Rather than focusing on individuals making judgements in isolation, our focus is on group deliberations about blameworthiness. This focus enables us to examine social influence in the context of blame. There are a couple of unique aspects to our study. First, we include a confederate in our deliberation groups, and the confederate makes scripted arguments regarding how the mitigating historicist narrative should be used (i.e., ignored or given great weight). Furthermore, following the deliberations we will separate participants from their groups so they can provide *private opinions* about blameworthiness, removed from the watchful eyes of fellow groups members. This procedure will enable us to explore whether and how the blame-mitigating force of historicist narratives—which is known to be moderated by a variety of factors [2, 3, 24]—is moderated by social influence. The separation of individual participants from their deliberation groups will enable us to see whether social influence truly changed people’s personal opinions. Finally, consistent with prior literature, we will consider mediation of historicist narrative effects via perceived control of self-formation and freedom of action. This will enable us to see whether social influence changes the mechanism by which historicist narratives mitigate blame.

In short, our research questions are: Can the mitigating force of a historicist narrative be strengthened or weakened by a peer’s arguments? If so, what intervening perceptions—i.e., freedom of action, control of self-formation—mediate this change in mitigating force?

## Method

### Participants

Participants were five-hundred thirty-two U.S.-based college students (three-hundred twenty-seven female; one participant did not provide information about biological sex) who earned credit in their Introductory Psychology course. For reasons that will be described below, data from twelve participants (six female, six male) were deleted prior to data analysis, leaving a final sample of five-hundred twenty. Data collection began in October 2018 and continued through November 2019.

### Procedure

We aimed to have groups of three participants (not including the confederate). For most groups, we met this goal (*N* = 140 groups). Due to no shows, some of our groups included just two participants (*N* = 56 groups). From the perspective of participants, they arrived in groups of three or four because we had an undergraduate female confederate arrive with the actual participants, wearing a backpack and pretending to be another participant from the Introductory Psychology class. Group members were escorted into our lab and were seated around a table that had two microphones on it. Another table containing a computer with a large monitor was a few feet from the group. An image of the setup can be seen at the bottom of this webpage: https://wordpress.lehigh.edu/blamelab/

After providing informed consent, participants were introduced to a study of “how groups reason and debate about blame and punishment” during which they would “listen to some information about a homicide case.” Although our goal was not to precisely replicate an actual criminal trial (indeed, we do not have the knowledge to do so), the general atmosphere created the sense of a “courtroom experience” in which evidence and arguments were presented and then “jurors”—our participants—were asked to deliberate about blameworthiness. Groups were randomly assigned to one of four conditions. In every condition, the experience began with an image on the computer monitor showing the mug shot of convicted double-murderer Robert Harris (discussed in [35]) next to an image of a lawyer who appeared to be making an argument. As these images were displayed, participants listened to a pre-recorded narrator who described the shockingly brutal manner in which Harris murdered two teen-aged boys. Full text of this narration—and all other narration described below—is available in the S1 File. For participants in the *deed only condition*, this was all they heard.

Other groups were in one of three *historicist narrative conditions*. Although these conditions differed in crucial ways that will be described shortly (the difference is introduced during deliberations), they were similar in one crucial respect. Specifically, in these conditions all groups saw the image on the computer shift to a different lawyer who appeared to be addressing a jury. Then, participants received a historicist narrative regarding Robert Harris. That is, they heard a different narrator tell the story of Robert Harris’s horrific childhood and how it deformed his moral character.

After hearing the appropriate information, each group was asked to deliberate about Harris’s blameworthiness. They learned that they would have ten minutes for discussion, at which point the experimenter would return to have them render some judgments about Harris. The confederate’s contributions during these deliberations constituted a crucial experimental manipulation. Specifically, during these conversations, the confederate interjected a scripted set of comments (the full script is available in [S1 File]). These scripted comments differentiated the three historicist narrative conditions from each other.

In the *narrative-neutral condition*, the confederate made non-committal statements (e.g., *I’ve never had to decide on a case like this one before*). The confederate followed this same neutral script in the deed only condition. Her goal in those conditions was to be non-influential. In the *narrative-ignore history condition*, the confederate argued that Harris’ life history should be ignored because it did not fully determine his horrific actions (e.g., *No matter what happened to him [in his childhood]*, *he wasn’t forced to be a murderer*; note that these arguments mirror the naturally occurring arguments in [19]). Finally, in the *narrative-affirm history condition*, the confederate argued that Harris’ history had such causal power that that Harris had little chance of turning out otherwise (e.g., *The first twenty years of his life were basically torture*. *It’s no wonder he became what he did*; these also mirror the naturally occurring arguments in [19]).

Following data collection, we deleted the data from four groups. One group was deleted because the confederate missed the session due to car trouble. Two groups were deleted—one from narrative–ignore history, one from narrative–affirm history—because the confederate was unable to interject her arguments into the group deliberations. In total, then, three groups were deleted due to confederate absence. A fourth group was deleted because a fire alarm went off during their deliberations. To their credit, all group members returned and completed the study, but a lot of time and activity had transpired between the deliberations and the dependent variables.

Audio recordings were made of all deliberations, and these were transcribed. After the study was completed, coders used the transcripts to count the number of times the confederate presented a scripted argument in each group. Two coders coded half of the groups and a different two coders coded the other half. Agreement within these two pairs of coders was *r* = .95 and *r* = .92, respectively. The number of scripted arguments delivered by the confederate in the narrative-ignore history (*M* = 3.21, *SD* = 1.25) and narrative-affirm history conditions (*M* = 3.17, *SD* = 1.36), where the confederate’s goal was to persuade, did not differ, *t*(188) = .15, *p* = .88, *d* = .03 (95% *CI*: -.38, .44). Similarly, the number of scripted arguments delivered by the confederate in the deed only (*M* = 1.90, *SD* = 1.06) and narrative-neutral conditions (*M* = 1.83, *SD* = .90), where the confederate’s goal was to remain neutral, did not differ, *t*(188) = .30, *p* = .77, *d* = .06 (95% *CI*: -.33, .45). A contrast pitting the narrative-ignore history and narrative-affirm history conditions against the deed only and narrative-neutral conditions revealed that the confederate made significantly more scripted arguments in the former two conditions than in the latter two, *t*(188) = 7.95, *p* < .001, *d* = 1.15 (95% *CI*: .84, 1.45). These analyses show that the confederate said more when she was supposed to be persuasive and said less when her role was to remain neutral. This suggests that the confederate was playing her role well.

Immediately following their deliberations, the groups were given a sheet of paper that explained how, in many states, an offender can receive a death sentence only if that sentence is unanimously agreed upon by the jury. Then, they read the following prompt:

*We ask you—as a “jury”—the following question: Does your group unanimously agree that Robert Harris should be sentenced to death for his crimes? That is, does every member of your group agree that Robert Harris should be put to death?*

The “jurors” circled “Yes” or “No” on the piece of paper. Overall, there was little support for the death penalty in this sample, with only 16% of groups assigning that penalty. Given that most Americans support the death penalty, this is an extremely low rate of support. This low rate of support for death did not differ as a function of which condition a group was in, *χ*^2^(3) = 3.30, *p* = .35. This suggests that death penalty decisions are an insensitive measure of blame in the present sample because many participants are categorically opposed to it. We assume they are categorically opposed because, if any offender deserves death, it is Robert Harris as portrayed in the deed only condition. Yet, even in that condition support for the death penalty was very low.

After making this collective decision, participants were escorted to separate rooms to provide their private opinions about Robert Harris. We separated participants to encourage them to report personal opinions rather than opinions that conform to those of fellow group members. That way, we could tell whether the confederate truly changed their minds. Participants were instructed not to put any self-identifying information on the survey. They were also assured that their fellow group members would not see their responses. They were instructed to drop their completed surveys into a locked metal ballot box located nearby and told that the ballot box would not be opened until all the data—from hundreds of participants—were collected.

These individual-level ratings were made on a seven-point scale ranging from 1 (*Strongly Disagree*) to 7 (*Strongly Agree*). The first ratings tapped our key variables of interest, blame-relevant emotional responses. As noted, we measured both blame and compassion. As defined above, our concept of blame involves hostile emotions felt toward the offender: Outrage, disgust, and so on. Accordingly, *moral outrage* was tapped by five items (e.g., *I feel moral outrage toward Robert Harris; I feel disgusted by Robert Harris*; *M* = 5.58, *SD* = 1.19, α = .86). *Compassion* was tapped by three items (e.g., *I feel compassion for Robert Harris*; *M* = 3.01, *SD* = 1.63, α = .87). The next ratings tapped perceptions of Robert Harris’ free will. Following [1], we created two different measures of free will. *Control of self-formation* tapped perceptions of whether Harris freely self-created his own personality (e.g., *Robert Harris had free will in terms of initially BECOMING the type of person he is*; *M* = 3.26, *SD* = 1.50, α = .87). *Freedom of action* tapped perceptions of whether, at the moment of action, Harris could have restrained himself (e.g., *Although Robert Harris had a strong inclination to be cruel*, *he could have used his human capacity for free will to inhibit such behavior*; *M* = 5.63, *SD* = 1.28, α = .90). Finally, to examine whether changes in moral outrage and/or compassion brought changes in punitive intentions (i.e., the behavioral component of blame; [1, 11]), we included items assessing the desire to see Robert Harris suffer *malicious punishments* (e.g., *I would be pleased to hear that the corrections officers in charge of Robert Harris were treating him very harshly*; *M* = 2.68, *SD* = 1.70, α = .94). All items are available in [S1 File].

Finally, participants completed some demographic items and then were debriefed.

## Results

Because individuals are nested within groups, data were analyzed using the Mixed Models feature of SPSS 28.0 with guidance from [36] and [37]. Our model treated “jury” as a random effect and examined the fixed effect of our experimental manipulation (i.e., deed only, narrative-neutral, narrative-ignore history, narrative-affirm history) with jury (*N* = 192) as the unit of analysis.

**Fig 1:**
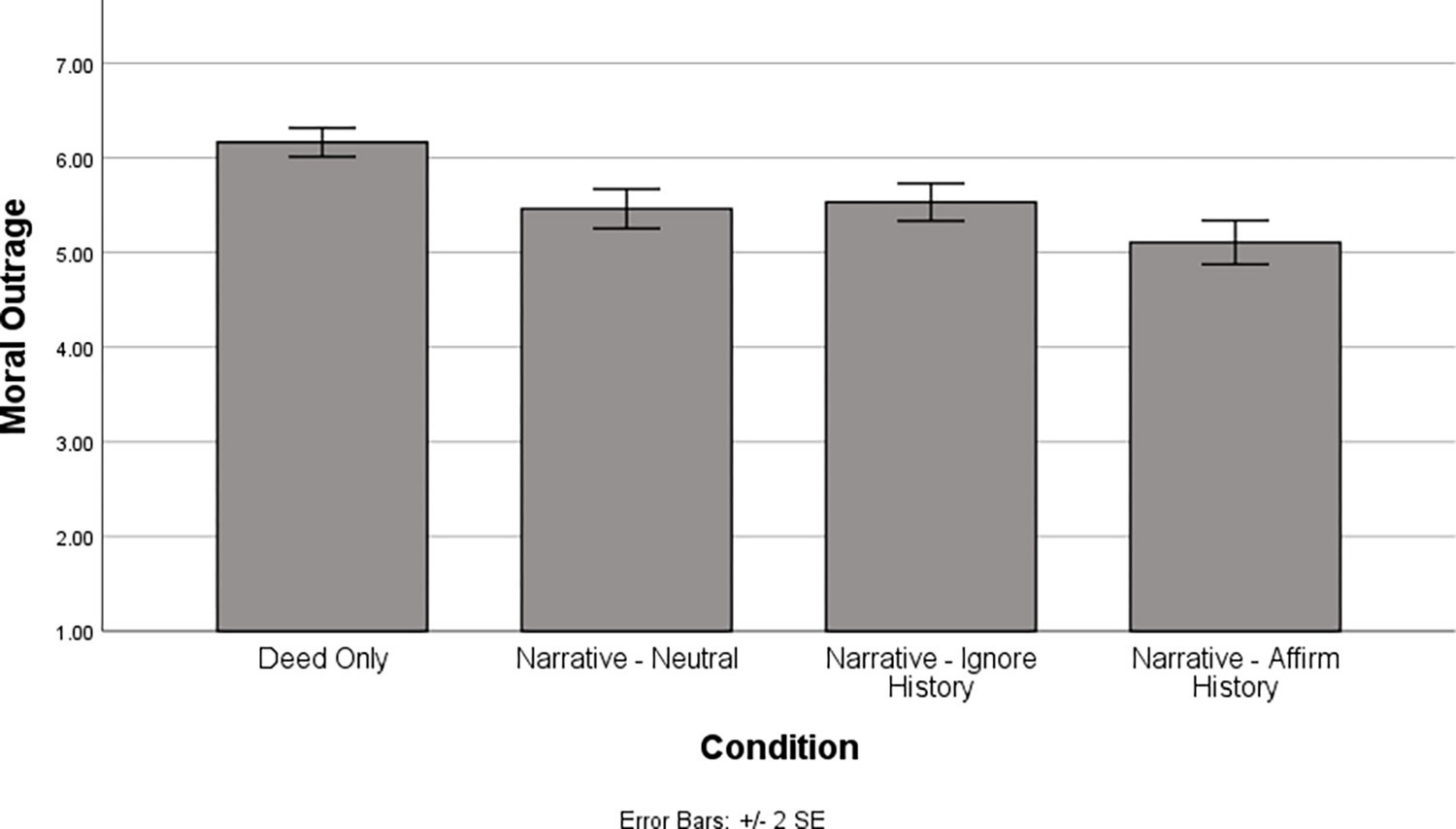
Moral outrage by condition.

First, we examined moral outrage. This revealed a main effect of condition, *F*(3, 184.035) = 15.27, *p* < .001. The pattern of results can be seen in Fig 1 and detailed information about simple effects is in Table 1. For our effect size measure, we computed Cohen’s *d* using formulas from [38]. When doing so, we used total variance (within- plus between-cluster) to compute the denominator based on the suggestion in [39] that total variance is most appropriate when the effect size pertains to an effect that is generally assessed at the individual rather than cluster-level in the relevant literature (which is the case for the blame mitigating impact of historicist narratives). Confidence intervals for the effect size were computed via bootstrapping (1,000 samples) using a program in *R* written by Dr. Chris Burke at Lehigh University. As can be seen in Fig 1 and Table 1, moral outrage was significantly higher in the deed only condition than in any of the narrative conditions. Thus, regardless of the arguments made by the confederate, the presence of a historicist narrative reduced moral outrage. Importantly, however, it was also the case that the narrative-affirm history condition resulted in significantly less moral outrage than all the other conditions. Thus, although the content of the historicist narrative was identical across all three narrative conditions, the presence of a confederate emphasizing the formative power of that history persuaded participants to reduce their moral outrage beyond what they did in the absence of such arguments. Interestingly, the narrative-neutral and narrative-ignore history conditions did not differ, suggesting that the confederate in the narrative-ignore history condition was unable to get participants become more outraged than they were in the absence of her arguments. This is an important finding given the ubiquity of naturally occurring ignore history arguments in prior work [19].

**Table 1:** Moral outrage as a function of condition: Simple effect comparisons.

	Deed Only	Narrative-Neutral	Narrative-Ignore History
Narrative-Neutral	*t*(182.52) = -4.50, *p* < .001 *d* = -.48 (-.68, -.29)		
Narrative-Ignore History	*t*(181.98) = -4.05, *p* < .001 *d* = -.44 (-.63, -.26)	*t*(178.20) = -.40, *p* = .69 *d* = -.04 (-.27, .17)	
Narrative-Affirm History	*t*(190.26) = -6.59, *p* < .001 *d* = -.72 (-.94, -.51)	*t*(186.19) = -2.14, *p* = .03 *d* = -.24 (-.47, -.003)	*t*(185.60) = -2.52, *p* = .01 *d* = -.28 (-.52, -.04)

*Note*. Numbers in parentheses reflect the 95% *CI* for Cohen’s *d*.

**Fig 2:**
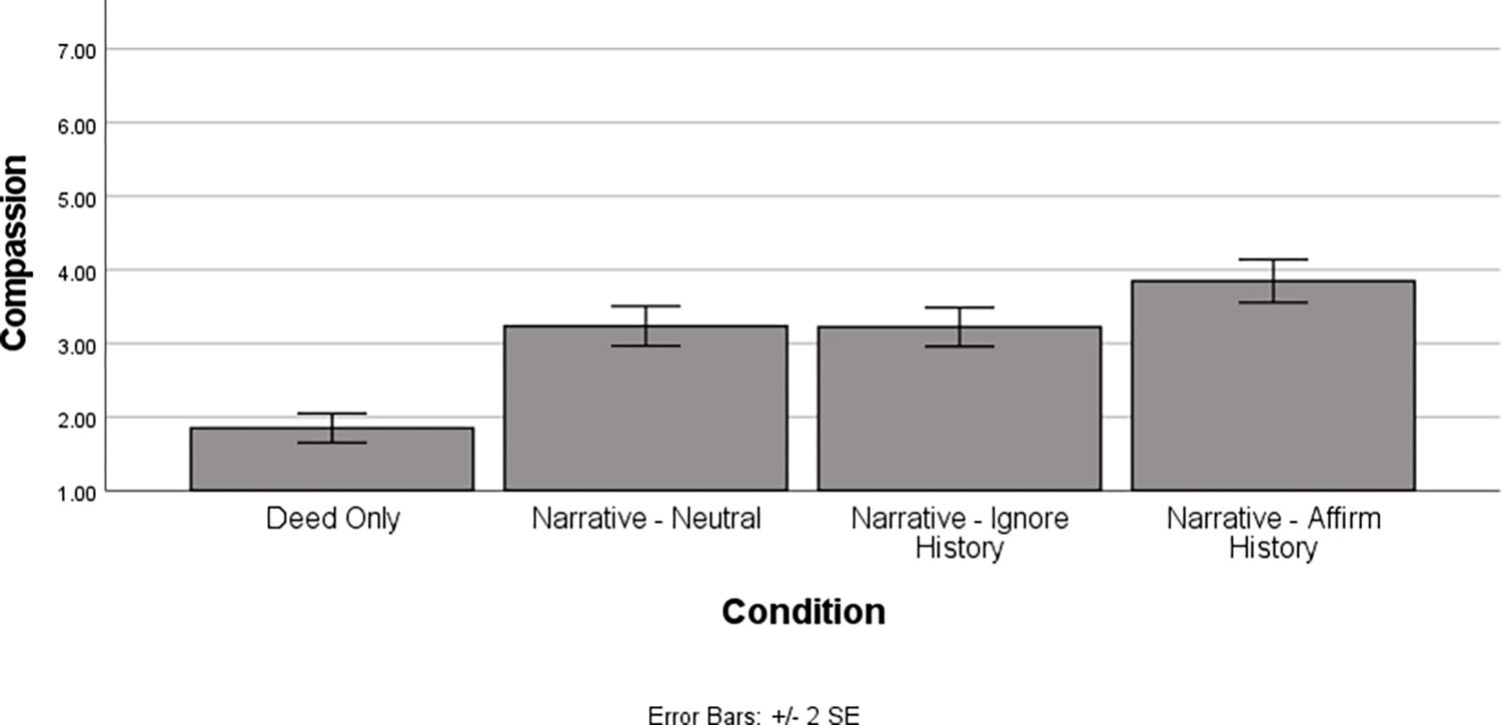
Compassion by condition.

Next, we examined compassion. This, too, revealed a main effect of condition, *F*(3, 186.54) = 33.83, *p* < .001. The pattern of results can be seen in Fig 2 and detailed information about simple effects is in Table 2. The results mirrored those for moral outrage. That is, compassion was significantly lower in the deed only condition than in any of the narrative conditions. Thus, regardless of the arguments made by the confederate during deliberations, the presence of a historicist narrative increased compassion compared to the deed only condition. Importantly, however, it was also the case that the narrative-affirm history condition produced significantly more compassion than all the other conditions. Thus, although the historicist narrative was identical across all three narrative conditions, the presence of the confederate arguing for the formative power of Harris’ life history led to increased compassion for Harris beyond levels that occurred in the absence of such a confederate. Interestingly, as with moral outrage, the narrative-neutral and narrative-ignore history conditions did not differ from each other, suggesting that the confederate in the narrative-ignore history condition was unable to convince participants to lessen the compassion they felt based on the narrative.

**Table 2:** Compassion as a function of condition: Simple effect comparisons.

	Deed Only	Narrative-Neutral	Narrative-Aggravating
Narrative-Neutral	*t*(184.97) = 6.76, *p* < .001 *d* = .73 (.52, .95)		
Narrative-Ignore History	*t*(184.42) = 6.69, *p* < .001 *d* = .73 (.53, .92)	*t*(180.45) = .01, *p* = .99 *d* = .00 (-.23, .25)	
Narrative-Affirm History	*t*(193.05) = 19.62, *p* < .001 *d* = 1.06 (.85, 1.28)	*t*(188.79) = 2.97, *p* = .003 *d* = .33 (.11, .56)	*t*(188.17) = 2.95, *p* = .004 *d* = .33 (.10, .57)

*Note*. Numbers in parentheses reflect the 95% *CI* for Cohen’s *d*.

In short, with the narrative-neutral condition as the baseline, the confederate in the narrative-affirm history condition was effective in boosting the power of the narrative to reduce moral outrage and to elicit compassion. Interestingly, relative to the narrative-neutral baseline, the confederate in the narrative-ignore history condition had no impact on others’ moral emotions (although she did have effects on other variables, as we will see shortly). Again, this lack of impact is arguably important given the ubiquity of naturally occurring ignore history arguments in prior work [19].

**Fig 3:**
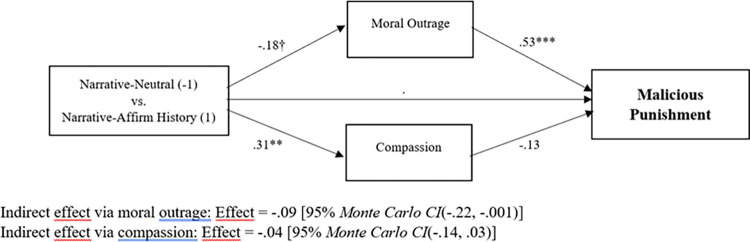
“Juries” in the narrative-affirm history condition show reductions in malicious punitive impulses, mediated by reduced moral outrage. Coefficients are unstandardized. †*p* = .05; **p < .01; ***p < .001.

Next, we computed a mediational model to examine whether, as in prior work centered on individual judgments [1, 11, 12], the moral-emotional changes among “juries” in the narrative-affirm history condition were associated with a reduction in malicious punishment intentions. We followed suggestions for testing multi-level mediation presented by Hayes and Rockwood [40] and we used Rockwood’s MLMED macro (downloadable at: https://njrockwood.com/mlmed) to implement the analysis. We used the narrative-neutral condition as our baseline control condition. The between-cluster effects—which treat the means of our “juries” as the unit of analysis—are shown in Fig 3. As already noted, moral outrage was lower in the narrative-affirm history condition than in narrative-neutral, *t*(88.40) = -1.97, *p* = .053. And, compassion was higher in narrative-affirm history than in narrative-neutral, *t*(92.83) = 2.77, *p* = .001. Furthermore, moral outrage was positively related to malicious punishment intentions, *t*(98.18) = 3.62, *p* < .001, whereas compassion was not related to malicious punishment intentions, *t*(94.29) = -1.11, *p* = .27. As can be seen in the statistical information beneath the model, the indirect effect traveling through moral outrage was significant, whereas the indirect effect traveling through compassion was not. This analysis is consistent with the possibility that a peer’s argument to give great weight to a historicist narrative reduces the malicious impulse to see the offender suffer, and this happens because the peer’s argument tempers feelings of moral outrage. Within-cluster effects—which indicate the association between each individual’s deviation from her/his cluster mean and the dependent variable—are also calculated by Rockwood’s MLMED macro. Because within-cluster effects have no relevance to present concerns (i.e., our manipulation was at the cluster-level), we report them in S1 File.

**Fig 4:**
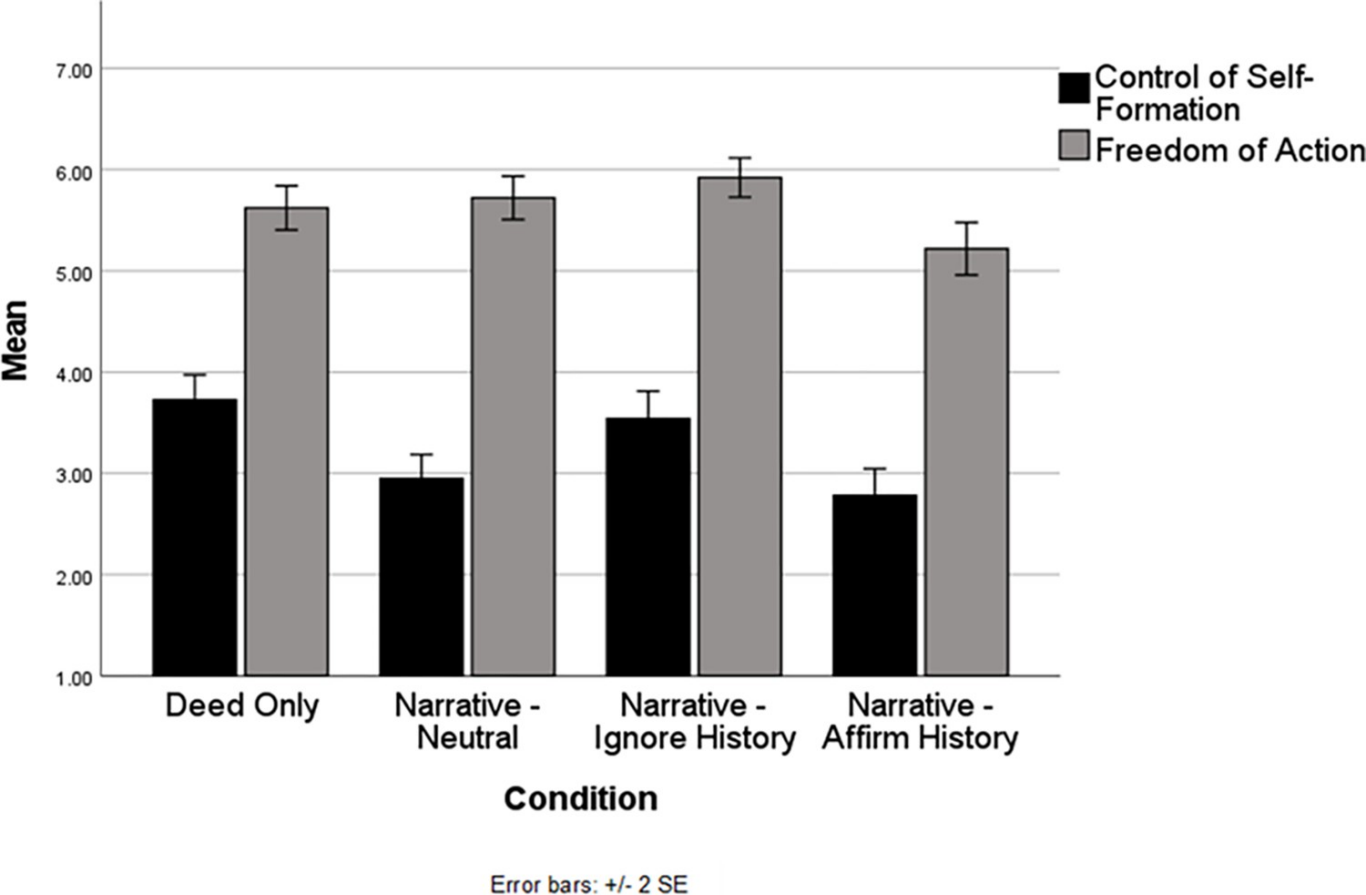
Freedom of action and control of self-formation by condition.

Next, we examined the impact of our manipulation on free will perceptions, perceptions which will later serve as potential mediators of the impact of our manipulation on outrage and compassion. We will discuss freedom of action and control of self-formation together because prior work suggests that historicist narratives typically diminish perceptions of the latter but only rarely impact the former (see review above). Our mixed model analysis of freedom of action revealed a main effect of condition, *F*(3, 181.40) = 6.50, *p* < .001, as did our analysis of control of self-formation, *F*(3, 181.45) = 10.38, *p* < .001. We will first discuss differences between the deed only and narrative-neutral conditions, as those are most analogous to the conditions of Gill and Cerce [1] (i.e., due to lack of an opinionated confederate). The pattern of results can be seen on the left half of Fig 4 and detailed information about simple effect tests is in Table 3. The results perfectly replicate Gill and Cerce: Perceptions of Robert Harris’ freedom of action do not differ across the deed only and narrative-neutral conditions, but perceived control of self-formation is lower in narrative-neutral as compared to deed only. These results replicate in the context of group deliberations the pattern that is typically found when individuals deliberate in isolation.

**Table 3:** Freedom of action and control of self-formation as a function of condition: Simple effect comparisons.

		Deed Only	Narrative-Neutral	Narrative-Ignore History
*Freedom of Action*	Narrative-Neutral	*t*(179.70) = .63, *p* = .53 *d* = -.07 (-.27, .13)		
	Narrative-Ignore History	*t*(179.01) = 1.86, *p* = .064 *d* = .20 (.01, .40)	*t*(174.07) = 1.23, *p* = .22 *d* = .13 (-.07, .33)	
	Narrative-Affirm History	*t*(189.27) = -2.51, *p* = .013 *d* = -.28 (-.52, -.04)	*t*(183.97) = -3.10, *p* = .002 *d* = -.35 (-.59, -.12)	*t*(183.23) = -4.29, *p* < .001 *d* = -.48 (-.71, -.27)
*Control of Self-Formation*	Narrative-Neutral	*t*(180.00) = -3.95, *p* < .001 *d* = -.42 (-.67, -.21)		
	Narrative-Ignore History	*t*(179.40) = .96, *p* = .34 *d* = -.10 (-.31, .12)	*t*(175.16) = 2.93, *p* = .004 *d* = .32 (.10, .54)	
	Narrative-Affirm History	*t*(188.17) = -4.76, *p* < .001 *d* = -.52 (-.74, -.30)	*t*(183.60) = -.87, *p* = .39 *d* = -.10 (-.30, .11)	*t*(182.95) = -3.72, *p* < .001 *d* = -.41 (-.65, -.19)

*Note*. Numbers in parentheses reflect the 95% *CI* for Cohen’s *d*.

Now, we can examine whether the introduction of an opinionated confederate changes that baseline pattern. First, in the narrative-ignore history condition, ratings of Harris’ freedom of action were not significantly different from narrative-neutral and were only marginally higher than deed only, whereas his control of self-formation was rated as significantly higher than in the narrative-neutral condition and equal to deed only. Thus, the confederate’s arguments in the narrative-ignore history condition were effective in completely erasing the diminished perceptions of control of self-formation usually elicited by historicist narratives (although, as noted above, this did not translate into increased moral outrage or decreased compassion). Results in the narrative-affirm history condition were different. There, ratings of Harris’ freedom of action became significantly lower than in both the deed only and narrative-neutral conditions, whereas ratings of his control of self-formation was rated as equal to narrative-neutral and significantly less than deed only condition. Thus, the narrative-affirm history condition altered the typical pattern associated with historicist narratives: Perceptions of both freedom of action control of self-formation were diminished relative to the deed only condition.

**Fig 5:**
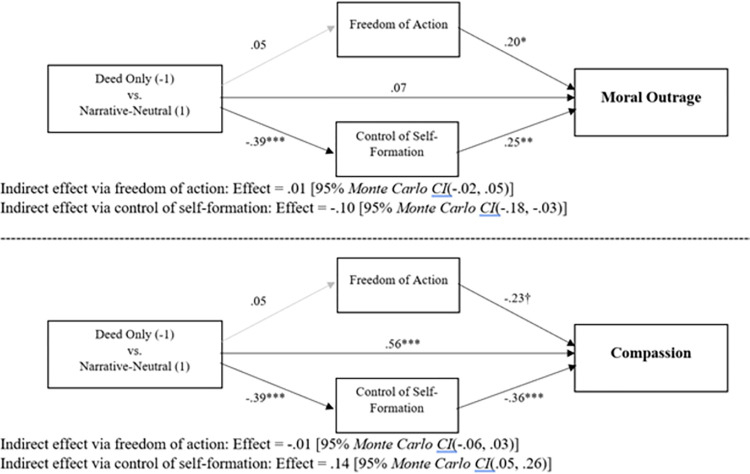
Mediation via freedom of action and control of self-formation of the differences in moral outrage and compassion between the deed only and narrative-neutral conditions. Coefficients are unstandardized. †*p* = .07; *p < .05; **p < .01; ***p < .001.

Finally, we turned to mediation of the moral-emotional changes (moral outrage, compassion) produced by our manipulation. As above, we followed suggestions regarding multilevel mediation in [40]. In line with prior work, we examined whether moral-emotional changes stemmed from altered perceptions of freedom of action or control of self-formation. First, we examined mediation of the difference between the deed only and narrative-neutral conditions. As noted, these conditions are analogous to the conditions in Gill and Cerce [1] due to the lack of an opinionated confederate. Thus, this test of mediation indicates whether we replicate in our group context the mediating mechanism uncovered by Gill and Cerce in their studies of isolated individuals. We begin with an analysis of moral outrage. The between-cluster effects are shown in top half of Fig 5. As can be seen there, and as described above, jury groups within the deed only and narrative-neutral conditions did not differ in their perceptions of Robert Harris’ freedom of action, *t*(267) = .65, *p* = .516. In contrast, the narrative-neutral condition resulted in lower perceptions of control of self-formation compared to deed only, *t*(267) = -4.52, *p* < .001. As noted, this perfectly replicates Gill and Cerce. Despite being unaffected by condition, perceived freedom of action was positively related to moral outrage, *t*(100.10) = 2.01, *p* = .047, as was control of self-formation, *t*(102.86) = 3.15, *p* = .002. As can be seen in the statistical information beneath the model, the indirect effect traveling through freedom of action was not significant, whereas the indirect effect traveling through control of self-formation was significant. This analysis shows that the mediating mechanism for historicist narratives—through diminished control of self-formation—is the same in the context of group deliberations as it is in the context of individual judgments, at least when the group context lacks a highly opinionated peer. As above, within-cluster effects are reported in S1 File.

Next, we analyzed mediation of differences in compassion between the deed only and narrative-neutral conditions. The between-cluster effects are shown in the bottom half of Fig 5. Of course, the effects of condition on freedom of action and control of self-formation are identical to those in the preceding paragraph. Here, however, perceived freedom of action was only marginally (negatively) related to compassion, *t*(97.37) = -1.83, *p* = .07. Perceived control of self-formation, however, showed a significant negative relation to compassion, *t*(99.94) = -3.54, *p* < .001. As can be seen in the statistical information beneath the model, the indirect effect traveling through freedom of action was not significant, whereas the indirect effect traveling through control of self-formation was significant. As above, within-cluster effects are reported in S1 File. In short, in the context of group deliberations that lack an opinionated confederate, historicist narratives reduce blame and increase compassion via the same mechanism—diminished perceptions of control of self-formation—that mediates their impact on individuals’ judgments.

**Fig 6:**
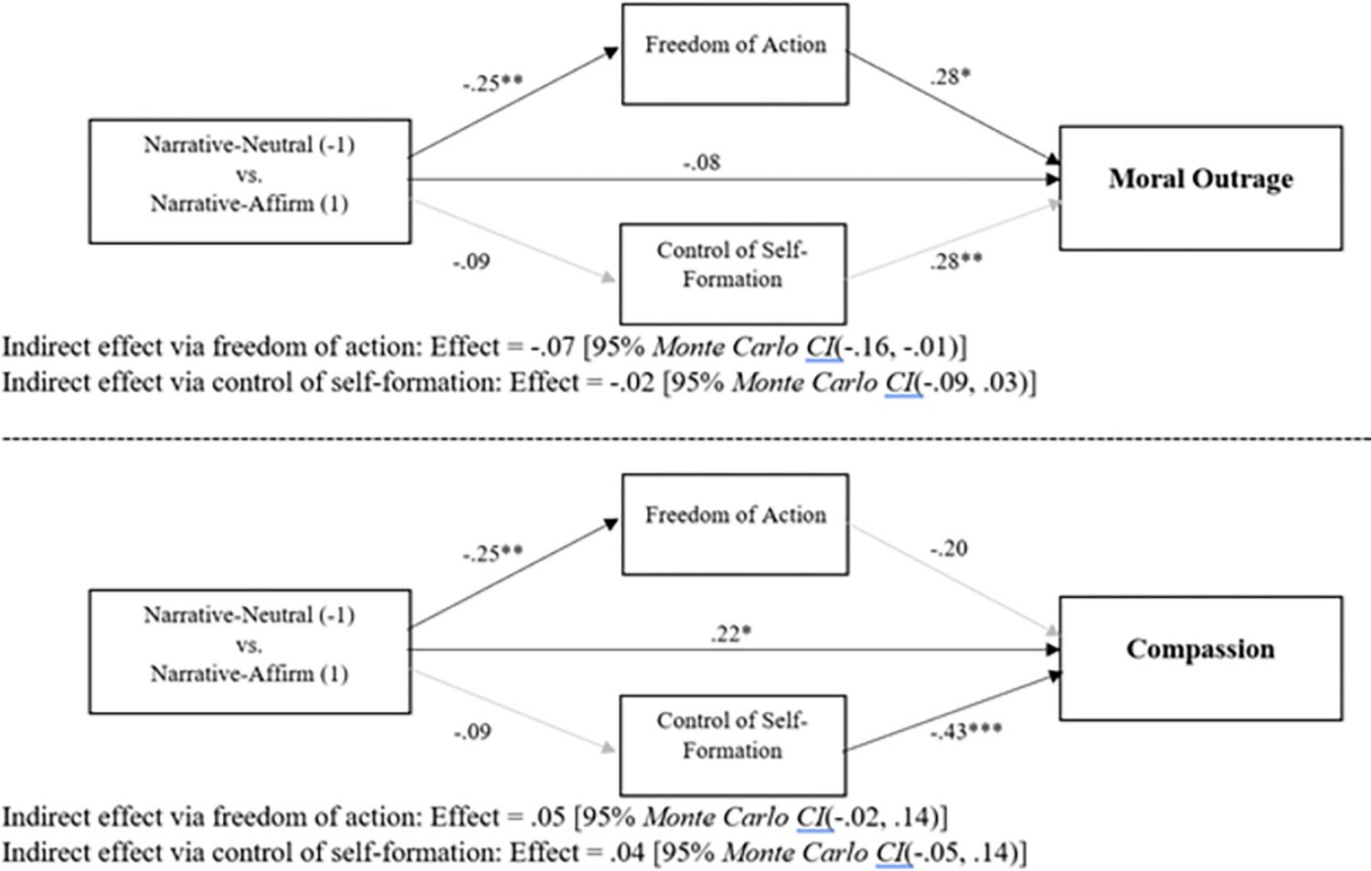
Mediation via freedom of action and control of self-formation of the differences in moral outrage and compassion between the narrative-neutral and narrative-affirm history conditions. Coefficients are unstandardized. *p < .05; **p < .01; ***p < .001.

Next, we turned to mediation of effects involving the narrative-affirm history condition, a condition which resulted in less moral outrage and more compassion than the other narrative conditions. We will focus here on comparing the narrative-affirm history to the narrative-neutral condition, thereby holding constant presentation of the narrative and varying only the content of the confederate’s arguments regarding that narrative. First, we examined differences in moral outrage. The between-cluster effects are shown in the top half of Fig 6. As can be seen there, and as reported above, perceived freedom of action was significantly lower in the narrative-affirm history condition than in narrative-neutral, *t*(87.59) = -2.92, *p* = .004. As noted above, perceived control of self-formation did not differ across the narrative-affirm history and narrative-neutral conditions, *t*(91.68) = -.86, *p* = .39. This implies that when the presentation of the narrative is held constant—as it is here—the impact of the confederate’s arguments regarding the narrative is uniquely captured by changes in perceived freedom of action. Perceived freedom of action was positively related to moral outrage, *t*(89.09) = 2.61, *p* = .01, as was perceived control of self-formation, *t*(85.11) = 3.02, *p* = .003. As can be seen in the statistical information beneath the model, the indirect effect traveling through freedom of action was significant, whereas the indirect effect traveling through control of self-formation was not. This mediation via reduced perceptions of freedom of action is a departure from what is typically found [1, 4, 27]. Instead, it is reminiscent of [3; Study 4], which reported mediation via of freedom of action when participants were encouraged to play the role of defense attorney. Thus, we now see two cases in which the mechanism by which historicist narratives mitigate blame shifts. Both cases involve participants receiving external encouragement to place great weight on the narrative. Thus, social influence seems to alter the inferences people draw from a historicist narrative. Notably, as can be seen in [S1 File], an analysis comparing the narrative-affirm history to the deed only condition yields the same pattern—mediation via freedom of action—as the analysis focused on the narrative-neutral condition. As above, within-cluster effects are reported in S1 File.

Next, we analyzed mediation of differences in compassion between the narrative-neutral and narrative-affirm history conditions. The between-cluster effects are shown in the bottom half of Fig 6. Of course, the effects of condition on freedom of action and control of self-formation are identical to those in the preceding paragraph. Perceived freedom of action was not significantly related to compassion, *t*(91.33) = -1.43, *p* = .16, whereas perceived control of self-formation was significantly negatively associated with compassion, *t*(87.13) = -3.58, *p* < .001. As can be seen in the statistical information beneath the model, neither indirect effect was significant. As in the analysis comparing narrative-affirm history to deed only (see [S1 File]), this analysis provides no clear evidence regarding mediation of the compassion effect. As above, within-cluster effects are reported in S1 File.

## Discussion

The blameworthiness of an offender is often discussed in groups. This is clearly the case in criminal justice settings, such as when capital cases require jurors to discuss aggravating and mitigating factors that might sway them towards or away from a death sentence [5–7]. It is also the case in various other settings of our lives, such as when we call our siblings to discuss the disappointing behavior of a family member, or when we spend the evening with our spouse discussing the latest outrageous act of a co-worker. Although judgments of blameworthiness are often produced via such group deliberations, the research literature overwhelmingly examines individuals assessing blameworthiness in isolation. Whereas such work is important for identifying the mechanisms by which blame is generated or mitigated, it leaves unexplored the social construction of blameworthiness. Furthermore, when social influence has been studied, the focus has been on peers’ persuasive efforts with no measure of the success of those efforts.

To address these gaps in the literature, the present study examined group deliberations about blameworthiness. Participants were placed in groups of two or three. Each group also included a confederate who followed a script. All groups learned about an offender who had committed a brutal double homicide. Some groups learned only about his horrific deed (deed only condition). Other groups additionally learned a historicist narrative regarding his brutal upbringing. We were interested in how participants utilized this potentially mitigating information about the offender’s history. In particular, we were interested in whether such utilization was impacted by the scripted arguments of the confederate. With regard to those arguments, our study design included three historicist narrative conditions: One in which the confederate remained neutral (narrative-neutral; the confederate was also neutral in the deed only condition), one in which the confederate argued that the historicist narrative should be ignored (narrative-ignore history), and one in which the confederate argued that the historicist narrative should be given great weight (narrative-affirm history). Our key dependent variables were moral outrage and compassion toward the offender.

Data were analyzed using a Mixed Models approach. Several noteworthy findings emerged. First, regardless of the confederate’s arguments, groups that received the historicist narrative expressed less moral outrage and more compassion than groups in the deed only condition. Yet, the impact of the narrative on both outrage and compassion was significantly stronger in the narrative-affirm history condition. Interestingly, the pattern of confederate influence was asymmetrical: The confederate in the narrative-ignore history condition had no influence on moral outrage or compassion. This suggests that, although arguments to ignore an offender’s unfortunate history can be common [19], they might not be very persuasive. A follow-up mediational analysis suggested that the reduced moral outrage in the narrative-affirm history was associated with decreased punitive desire to see the offender suffer maltreatment in prison.

We also examined mediation of the impact of historicist narratives on moral outrage and compassion. Following prior work [1], we focused on two different types of free will belief: Freedom of action (“he could’ve chosen not to commit the act”) and control of self-formation (“his moral character was freely self-created”). We began by asking whether the typical mechanism connecting historicist narratives to outrage and compassion—i.e., reduced perceptions of control of self-formation—is operative in the context of group deliberations (as opposed to individual judgments, as in prior work). Indeed, group deliberations create a context in which participants could spontaneously offer a variety of novel arguments regarding the implications of a historicist narrative. Plausibly, these arguments could lead to the impact of historicist narratives being mediated by different (or additional) mechanisms, as participants’ naturally occurring arguments alter the inferences drawn from the narrative.

We began by looking at mediation of the differences in moral outrage and compassion between the deed only and narrative-neutral conditions. These conditions are most comparable to prior work on individual judgments of blame [1] because they lack an opinionated confederate. Following prior work, we treated free will beliefs—perceived freedom of action, perceived control of self-formation—as potential mediators. Results revealed perfect replication of what has been found by prior work examining individual blame judgments. That is, groups in the deed only and narrative-neutral conditions did not differ in their assessments of the offender’s freedom of action. They did, however, differ in their assessments of his control of self-formation, with groups in the narrative-neutral condition perceiving less control of self-formation than groups in the deed only condition. This diminished perception of control of self-formation mediated both the reduced moral outrage and the increased compassion found in the narrative-neutral condition. Thus, at least in the absence of an opinionated confederate, groups seem to produce similar patterns of judgments in response to historicist narratives as do individuals.

Next, we conducted mediation analyses to explore why the narrative-affirm history condition was particularly effective at reducing moral outrage and increasing compassion. We focused on the differences between the narrative-affirm history versus narrative-neutral, thus holding constant the presentation of the narrative but varying confederate arguments. The narrative-affirm history condition produced significantly less outrage and significantly more compassion than the narrative neutral condition. The mediation analysis pointed to a novel conclusion: The presence of a confederate urging others to utilize the historicist narrative reduced moral outrage by reducing perceptions of the offender’s freedom of action. This differs from the typical mechanism, mentioned above, involving control of self-formation. Yet, it is similar to what was uncovered by Gill and Ungson [3] in a study where participants were encouraged to adopt the mindset of a defense attorney. In that case, the “defense attorneys” presented with a historicist narrative showed a particularly strong mitigating effect of that narrative, with mitigation being mediated by, as in the present analysis, reduced perceptions of offender freedom of action. One obvious similarity between the narrative-affirm history condition of the present study and the defense attorney condition of Gill and Ungson is that, in both cases, participants were receiving social encouragement or pressure (from the experimenter in Gill and Ungson and from the confederate in the present study) to extract as much blame mitigating meaning from the historicist narrative as possible. Arguably, such encouragement or pressure could lead participants to draw inferences from historicist narratives—i.e., inferences about diminished freedom of action—that they do not make without such pressure or encouragement. Notably, a mediation analysis that substituted the deed only condition for the narrative-neutral condition found identical results to those just described.

We believe inferences of diminished freedom of action when confronted with a historicist narrative are reasonable, even if not typically made. That is, we do not assume that such inferences reflect an indefensible bias in reasoning. In fact, in discussions of our work with colleagues we have often been asked: How is freedom of action possible for a person whose moral character has been deformed by an unfortunate life history? Indeed, to judge that a person with an unfortunate life history has an unchosen, deformed moral character but that he nevertheless maintains freedom of action does involve some questionable assumptions. As articulated by Gill and Cerce, one possibility for explaining why lay people can believe that freedom of action is intact even in those with a damaged moral character is that “laypeople endorse a libertarian conception of free will (see Fischer et al., 2007). Such a conception asserts that human consciousness grants a special power to ‘transcend causality’ and to make free choices no matter what has happened previously. Most philosophers admit that such a view is difficult to defend (Mele, 2008), but laypeople might nevertheless endorse it” [1, pg. 364)]. One way of viewing the freedom of action results in the present study (and in [3]) is that, under social encouragement or pressure to extract maximum mitigating meaning from historicist narratives, participants come to realize the dubious nature of claiming that a person’s character has been deformed yet that he is still free to make choices at odds with that deformed character. Indeed, such a view is dubious because it assumes that choices stem from something other than “how one is, mentally speaking” (i.e., one’s character; [41; pg. 6]), and it’s hard to pinpoint what that “something else” is. Clearly, more research is needed to explore the complexities of how laypeople think about free will and whether further evidence can be produced in support of the idea that historicist narratives will generally reduce perceived freedom of action when people are motivated to extract as much mitigating meaning from the narrative as possible.

The present results contribute new knowledge concerning issues raised in our Introduction. In the Introduction we reviewed evidence suggested that the impact of historicist narratives on mitigation is malleable. Of relevance here, we cited prior work suggesting that their mitigating impact is strengthened when they are paired with a scientifically-grounded theoretical rationale that clarifies how a difficult history impairs character development [24]. The present work shows that social influence from peers is another factor that moderates the impact of historicist narratives on mitigation. Peers are especially effective, we found, when they argue in favor of (rather than against) strong mitigation based on the narrative. Notably, in the present study the peer’s arguments centered on how an unfortunate history diminished the offender’s freedom and control vis-à-vis action and character development. At an abstract level, the effectiveness of these peer arguments might depend on a similar mechanism to the mechanism involved when a scientifically-grounded theoretical rationale is provided [24]: Both the peer arguments and the theoretical rationale *underline the causal power of offender history* in shaping the offender. This point harkens back to Gill and Cerce’s [1] argument that offender history might matter to the extent that it *explains character development* (as opposed to merely portraying suffering). Future work should explore other potential peer arguments that might moderate the mitigating power of historicist narratives. Furthermore, future work should also explore social influence on the mitigating impact of biological narratives, which also have a malleable degree of impact on mitigation (see Introduction).

Although we think the present work makes important contributions to our understanding of social influence in blame deliberations and of the mechanisms by which historicist narratives mitigate blame, of course there are also limitations. Most significantly, we do not know how well the present results would generalize to actual jury deliberations or to real-world assessments of blameworthiness in non-legal contexts. Mock jury studies differ in many ways from the situation of an actual jury. Of course, although we cannot assume generalization, we should also refrain from excessive scepticism about it, as blame effects in the lab can be similar to those in the actual courtroom (e.g., see Introduction to [27]). Another limitation is that the present study assessed punishment of the offender only using items that expressed a desire to the see the offender suffer. An examination of how historicist narratives impact a broader range of responses to offenders is needed. The reason is that offender suffering is only one of many possible goals behind people’s responses to an offender. That is, people sometimes punish based on retributive goals (“an eye for an eye”) but other times undertake responses to prompt moral change in the offender (rehabilitation), to remove the offender from society but in a way that does not aim to make him suffer (incapacitation), or to deter others from behaving like the offender (deterrence) [42]. Notably, prior work suggests that historicist narratives remove the desire to see the offender suffer without removing the desire to pressure or encourage moral change in the offender [1]. Nevertheless, there is surely more to learn about how historicist narratives shift punishment goals away from retribution/offender suffering and toward alternatives.

Many possibilities for future research are suggested by the present approach. Most fundamentally, the blame literature would benefit from additional work on persuasion in the context of blaming. As noted, blameworthiness is often discussed in groups. Also, excessive blaming is problematic in a variety of ways [43–48]. Thus, a useful area for future work would be to identify how individuals can successfully persuade others to blame more temperately. Is it most effective for the persuader to emphasize the importance of mitigating factors, as in the present work? Is it effective for the persuader to appeal to moral values such as compassion and mercy, invoked without reference to any concrete mitigating information? Is it effective for the persuader to highlight the inefficacy of harsh blame, pointing out how intense blame can often produce undesired outcomes [43–48]? Is it effective for the persuader to emphasize how excessive blaming can make the blamer appear immature or incompetent [49]? Answers to all these questions would be informative about how to increase rates of wise blaming in the world.

## Conclusion

The present study joins a small number of studies that examine social influence in the context of blaming. Our results showed that a confederate’s arguments to place great weight on an offender’s historicist narrative reduced moral outrage and increased compassion beyond mere presentation of the narrative. The reduction in moral outrage accomplished by the confederate’s arguments mediated a reduction in spiteful punitiveness toward the offender. Interestingly, and conversely, the confederate’s arguments to ignore the narrative had no impact on outrage or compassion (although they did affect perceptions of the offender’s free will in the expected direction). Future research is needed to better understand the role of persuasion in facilitating temperate blaming.

## Supporting information

S1 File (DOCX)
