# Supplementary material for: What do other people think he deserves? Social influence on utilization of mitigating information regarding a violent offender’s unfortunate life history
Source: PLoS One. 2023 Nov 17;18(11):e0291729. doi: 10.1371/journal.pone.0291729 (PMC10656008; doi:10.1371/journal.pone.0291729)

**Gill and Zungu, What do other people think he deserves?**

**SUPPLEMENTAL MATERIALS**

**NARRATION REGARDING ROBERT HARRIS CASE**

**DEED (heard by groups in all four conditions)**

On July 5, 1978, John Mayeski and Michael Baker were sitting in the parking lot of a fast-food restaurant eating lunch. Mayeski and Baker lived on the same street and were best friends. They were on their way to a nearby lake for a day of fishing. Little did they know the horror they were about to experience at the hands of the defendant, Robert Alton Harri.

At the other end of the parking lot, Robert Harris and his brother Daniel were trying to hotwire a car when they spotted the two boys. The Harris brothers were planning to rob a bank and did not want to use their own car. When Robert Harris could not start the car, he pointed to the car where the 16-year-olds were eating and said to Daniel, "We'll take this one."

Robert Harris pointed a gun at Mayeski, crawled into the back seat, and told him to drive east. Daniel Harris followed in the Harrises' car. When they reached a canyon area, Robert Harris told the youths he was going to use their car in a bank robbery and assured them that they would not be hurt. Robert Harris yelled to Daniel to get the .22 caliber rifle out of the back seat of their car.

The boys said that they would wait in the canyon area until the Harrises were gone and then walk into town and report the car stolen. Robert Harris agreed.

As the two boys walked away, Harris slowly raised the gun and shot John Mayeski in the back. Mayeski yelled: "Oh, God," and slumped to the ground. Harris chased Michael Baker down a hill into a little valley and shot him four times. Mayeski was still alive when Harris climbed back up the hill. Harris walked over to the boy, knelt down, put the Luger to his head and fired. Daniel Harris said that his brother Robert was "swinging the rifle and pistol in the air and laughing. His laugh made blood and bone freeze in me."

Robert Harris drove the boys’ car to a friend's house where he and Daniel were staying. Harris walked into the house, carrying the weapons and a bag containing the remainder of the slain youths' lunch. Then, about 15 minutes after he had killed the two 16-year-old boys, Robert Harris took the food out of the bag and began eating a hamburger. He offered his brother an apple turnover, and Daniel became nauseated and ran to the bathroom. "Robert laughed at me," Daniel said. "He said I was weak; he called me a sissy and said I didn't have the stomach for it."

Harris was in an almost lighthearted mood. He smiled and told Daniel that it would be amusing if the two of them were to pose as po­lice officers and inform the parents that their sons were killed.

Later, as they prepared to rob the bank, Harris pulled out his gun, noticed blood stains and remnants of flesh on the barrel as a result of the point-blank shot, and said, "I really blew that guy's brains out." And then, again, he started laughing.

Prior to murdering Mayeski and Baker, Robert Harris was arrested twice for torturing animals and was convicted of manslaughter for beating a neighbor to death after a dispute.

It is imperative that Robert Harris be punished severely for this double-murder. He is an evil man and a danger to society.

**HISTORICIST NARRATIVE (heard by participants in all three narrative conditions)**

Psychologists have sought to understand the making of Robert Harris by interviewing people who knew him well. They have painted a heart-wrenching portrait of how an innocent child becomes an evil adult.

Harris was born Jan. 15, 1953, hours after his mother was kicked in the stomach. She was 6 months pregnant and her husband came home drunk and accused her of in¬fidelity. He kicked her. She began hemorrhaging, and he took her to the hospital. Robert was born that night. Because of the premature birth, he was a tiny baby; he was kept alive in an incubator for months at the hospital.

All of the Harris children had monstrous childhoods. But even in the Har¬ris family the abuse Robert was subjected to was unusual. Before their mother died last year, Barbara Harris (Robert’s sister) said her mother talked incessantly about Robert's early years. She felt guilty that she was never able to love him; she felt partly responsible for his crimes.

The injury Robert's mother suffered as a result of the birth, and the constant abuse she was subjected to by her husband, turned her against her son. She began to blame all of her problems on Robert, and she grew to hate the child. Harris’s sister Barbara states: "I remember one time we were in the car and Mother was in the back seat with Robbie in her arms. He was crying and my father threw a glass bottle at him, but it hit my mother in the face. The glass shat¬tered and Robbie started screaming. I'll never forget it" … "Mom’s face was all pink, from the mixture of blood and milk. She ended up blaming Robbie for all the hurt, all the things like that. She felt helpless and, even though he was just a baby, he was someone to vent her anger on."

Harris had a learning disability and a speech problem, but there was no money for therapy. When he was at school he felt stupid and classmates teased him, his sister said, and when he was at home he was abused. "He was the most beautiful of all my mother's children; he was an angel," she said. "He would just break your heart. He wanted love so bad he would beg for any kind of physical contact. He'd come up to my mother and just try to rub his little hands on her leg or her arm. He just never got touched at all. She'd just push him away or kick him. One time she bloodied his nose when he was trying to get close to her." While sharing this story, Barbara Harris put her head in her hands and cried softly. "The sad thing is he was the most sensitive of all of us. When he was two and we all saw `Bambi,' he cried and cried when Bambi's mother was shot. Everything was pretty to him as a child; he loved animals. But all that changed; it all changed so much."

At age 14, Harris was sentenced to a federal youth detention center for car theft. He was one of the youngest inmates there, Barbara Harris said, and he grew up "hard and fast." Harris was raped several times, his sister said, and he slashed his wrists twice in suicide attempts.

The centers were "gladiator schools," Barbara Harris said, and Har¬ris learned to fight and to be mean. By the time he was released from federal prison at 19, all his problems were accentuated. Everyone in the family knew that he needed psychiatric help. The child who had cried at the movies when Bambi's mother dies had evolved into a man who was arrested several times for abusing an¬imals. He killed cats and dogs, Daniel said, and laughed while tortur¬ing them with mop handles, darts and pellet guns. Once he stabbed a prize pig more than 1,000 times.

"The only way he could vent his feelings was to break or kill something," Barbara Harris said. "He took out all the frustrations of his life on animals. He had no feeling for life, no sense of remorse. He reached the point where there wasn't that much left of him." She stared out a window. "Well, I still remember the little boy who used to beg for love, for just one pat or word of kindness."

**CONFEDERATE ARGUMENTS DURING DELIBERATIONS**

**Neutral Confederate (Deed Only condition; Narrative—Neutral condition)**

i. What he did was so awful…

ii. I’ve never had to decide on a case quite like this before …

iii. Obviously, he needs to be in prison, but I just don’t know what level of harshness is right…

**Narrative—Affirm History Condition**

i. Guys, we heard about his life…c’mon…was there any chance he could turn out to be

anything other than a mean, angry person?

ii. Clearly, he is an awful person…but was that his original goal in life? Did his life

experiences leave much chance of him turning out good?

iii. The first 20 years of his life were basically torture. It’s no wonder he became what he did.

**Narrative—Ignore History Condition**

i. He took a LONG time to kill those boys. He had lots of time to think about what he was

doing…it was highly deliberate.

ii. Yes, his life was horrible, but he STILL knew that killing people is forbidden. He chose to

kill KNOWING that it is forbidden.

iii. No matter what has happened to them, everyone can still make a choice about how they act.

He wasn’t forced to be a murderer.

**DEPENDENT VARIABLES**

**MORAL OUTRAGE**

- I blame Robert Harris for his violent crimes.
- I feel moral outrage toward Robert Harris.
- I feel hatred toward Robert Harris.
- I feel disgusted by Robert Harris.
- I have powerful feelings of dislike toward Robert Harris.

**COMPASSION**

- I feel compassion for Robert Harris.
- I feel sympathy for Robert Harris.
- I feel sorry for Robert Harris.

**CONTROL OF SELF-FORMATION**

- Robert Harris had free will in terms of initially BECOMING the type of person he is.
- Throughout his life, Robert Harris was always in control of his personality development.
- Robert Harris’s negative traits are purely a result of him freely choosing to become who

he currently is.

**FREEDOM OF ACTION**

- By using his human capacity for free will, Robert Harris could have chosen NOT to be cruel to the boys he murdered.
- It was possible for Robert Harris to use his free will to overcome his negative impulses and behave more appropriately.
- Although Robert Harris had a strong inclination to be cruel, he could have used his human capacity for free will to inhibit such behavior.

**MALICIOUS PUNISHMENT**

- It would be great to learn that Robert Harris was violently attacked by another inmate during his time in prison.
- I would be happy to hear that Robert Harris’s fellow inmates stole personal possessions from him.
- I would be pleased to hear that the corrections officers in charge of Robert Harris were treating him very harshly.
- I hope that another inmate will “put Robert Harris in his place,” and make him look like a weak loser in front of everyone.

**ADDITIONAL STATISTICAL ANALYSES**

**Multi-Level Mediation Models: Within-Cluster Results**

**I. Conditions: Deed Only, Narrative-Neutral; DV = moral outrage; Mediators = freedom of action, control of self-formation**

The within-cluster results indicated a significant positive relation between freedom of action and blame within clusters, *t*(172.17) = 2.05, *p* = .042 (*B* = .13), but no such relation between control of self-formation and blame within clusters, *t*(172.17) = .72, *p* = .47 (*B* = .04).

**II. Conditions: Deed Only, Narrative-Neutral; DV = compassion; Mediators = freedom of action, control of self-formation**

The within-cluster results indicated no relation between freedom of action and compassion within clusters, *t*(169.21) = -1.23, *p* = .22 (*B* = -.09), but a significant negative link between control of self-formation and compassion within clusters, *t*(169.21) = -3.55, *p* < .001 (*B* = -.26).

**III. Conditions: Deed Only, Narrative-Affirm History; DV = moral outrage; Mediators = freedom of action, control of self-formation**

The within-cluster results indicated a marginal positive relation between freedom of action and moral outrage within clusters, *t*(156.66) = 1.97, *p* = .051 (*B* = .12), and a significant positive link between control of self-formation and moral outrage within clusters, *t*(157.31) = 2.90, *p* = .004 (*B* = .18).

**IV. Conditions: Deed Only, Narrative-Affirm History; DV = compassion; Mediators = freedom of action, control of self-formation**

The within-cluster results indicated no relation between freedom of action and compassion within clusters, *t*(158.89) = -.51, *p* = .61 (*B* = -.04), but a significant negative link between control of self-formation and compassion within clusters, *t*(159.51) = -4.57, *p* < .001 (*B* = -.37).

**V. Conditions: Narrative-Neutral, Narrative-Affirm History; DV = moral outrage; Mediators = freedom of action, control of self-formation**

The within-cluster results indicated a significant positive relation between freedom of action and moral outrage within clusters, *t*(155.86) = 3.35, *p* = .001 (*B* = .23), and a significant positive link between control of self-formation and moral outrage within clusters, *t*(156.60) = 2.12, *p* = .04 (*B* = .15).

**VI. Conditions: Deed Only, Narrative-Affirm History; DV = compassion; Mediators = freedom of action, control of self-formation**

The within-cluster results indicated no relation between freedom of action and compassion within clusters, *t*(158.29) = -1.25, *p* = .21 (*B* = -.11), but a significant negative link between control of self-formation and compassion within clusters, *t*(159.02) = -3.43, *p* < .001 (*B* = -.43).

**Mediation: Narrative-Affirm History vs. Deed Only – Impacts on Outrage and Compassion Mediated Via Freedom of Action and Control of Self-Formation**

Here, we focus on differences in moral outrage between narrative-affirm history and deed only. This analysis tests whether the typical mechanism of historicist narratives—i.e., control of self-formation—changes under the social pressure/encouragement from the confederate to utilize the narrative. The between-cluster effects are shown in the top half of the Figure below. As can be seen there, and as reported above, perceived freedom of action was significantly lower in the narrative-affirm history condition than in deed only, *t*(92.97) = -2.34, *p* < .05. This is different from what we found when we compared narrative-neutral to deed only, which revealed no difference in freedom of action perceptions. Apparently, then, a historicist narrative can bring reductions in perceived freedom of action when a peer encourages one to appreciate the causal power of the offender’s history. Perceived control of self-formation was also lower in narrative-affirm history than in deed only, *t*(89.53) = -4.84, *p* < .001, and the size of the coefficient was over twice as large as the coefficient for freedom of action. Thus, the impact of a historicist narrative on perceived control of self-formation continues to be pronounced in this novel condition. Perceived freedom of action was positively related to moral outrage, *t*(92.07) = 3.23, *p* = .002, whereas, surprisingly, perceived control of self-formation was not, *t*(96.01) = 1.52, *p* = .13. As can be seen in the statistical information beneath the model, the indirect effect traveling through freedom of action was significant, whereas the indirect effect traveling through control of self-formation was not. This mediation via reduced perceptions of freedom of action is a departure from what is typically found [1, 4, 24]. Instead, it is reminiscent of [3; Study 4], which reported mediation via of freedom of action when participants were encouraged to play the role of defense attorney. Thus, we now see two cases in which the mechanism by which historicist narratives mitigate blame shifts. Both cases involve participants receiving external encouragement to place great weight on the narrative. Thus, social influence seems to alter the inferences people draw from a historicist narrative. Within-cluster effects are reported above.

Next, we analyzed mediation of differences in compassion across the narrative-affirm history and deed only conditions. The between-cluster effects are shown in the bottom half of the Figure below. Of course, the effects of condition on freedom of action and control of self-formation are identical to those in the preceding paragraph. Perceived freedom of action, however, was only marginally (negatively) related to compassion, *t*(93.99) = -1.94, *p* = .051, whereas, surprisingly, perceived control of self-formation was not significantly associated with compassion, *t*(98.12) = -1.44, *p* = .15. As can be seen in the statistical information beneath the model, neither indirect effect was significant. This means that we lack clear evidence of what mediates the impact of the narrative-affirm history condition on compassion. As above, within-cluster effects are reported above.


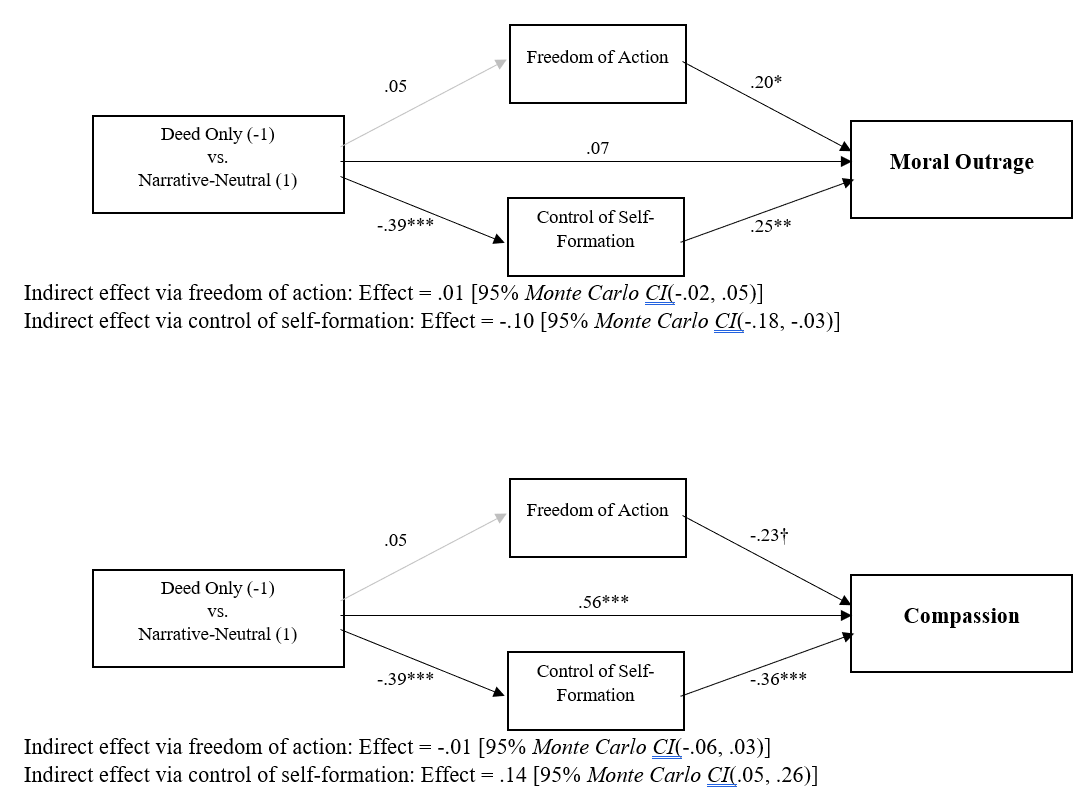

Supplement: S1 File — (DOCX) [file pone.0291729.s001.docx]
